# Emergency Department Provider Utilization in Rural and Urban Settings: Roles of NPs, PAs, and Physicians

**DOI:** 10.1093/geroni/igaf122.2586

**Published:** 2025-12-31

**Authors:** Nasim Ferdows

**Affiliations:** Northeastern University, Boston, Massachusetts, United States

## Abstract

Emergency departments (EDs) are essential access points in the U.S. healthcare system, with increasing reliance on nurse practitioners (NPs) and physician assistants (PAs) due to rising patient volumes and physician shortages. However, little is known about how provider utilization varies by patient demographics, visit acuity, and geographic location. Using data from the 2017–2021 National Hospital Ambulatory Medical Care Survey, we examined provider types across 83,282 ED visits, categorized as ED physician only (74.0%), NP/PA with a physician (14.4%), and NP/PA without a physician (11.6%). Younger patients (< 15 years) were more likely to see NP/PAs without a physician (13.6%), while older adults (≥65 years) were more often treated by ED physicians only (78.9%). Non-Hispanic Black patients had the highest likelihood of NP/PA involvement (16.4%). Semiurgent/nonurgent visits had significantly higher odds of NP/PA-only care (OR: 5.39; 95% CI: 4.80–6.04), while immediate/emergent cases were primarily managed by ED physicians (78.8%). Rural EDs had higher NP/PA-only involvement (13.5%) compared to urban EDs (9.8%; OR: 1.08; 95% CI: 1.01–1.16). Public insurance holders were more likely to see NP/PAs with a physician, while self-pay patients had higher odds of NP/PA-only care (OR: 1.35; 95% CI: 1.25–1.45). These findings highlight workforce distribution patterns in EDs, with NP/PAs playing a greater role in rural and lower-acuity settings. Understanding these trends is critical for workforce planning and optimizing ED resource allocation.

